# Kinetic study of removal heavy metal from aqueous solution using the synthetic aluminum silicate

**DOI:** 10.1038/s41598-020-67720-0

**Published:** 2020-07-02

**Authors:** Manuel Alejandro Treto-Suárez, Julio Omar Prieto-García, Ángel Mollineda-Trujillo, Emilio Lamazares, Yoan Hidalgo-Rosa, Karel Mena-Ulecia

**Affiliations:** 1 Doctorado en Fisicoquímica Molecular, Universidad Andres Bello, Ave. República 275, 8320000 Santiago, Chile; 2 Departamento de Química y Farmacia, Universidad Central “Marta Abreu” de las Villas, Carretera de Camajuani km 5, 50100 Villa Clara, Cuba; 3 Biotechnology and Biopharmaceutical Laboratory, Pathophysiology Department, School of Biological Sciences, Universidad de Concepción, Victor Lamas 1290, P.O. Box 160-C, 4030000 Concepción, Chile; 4 Departamento de Ciencias Biológicas y Químicas, Facultad de Recursos Naturales, Universidad Católica de Temuco, Ave. Rudecindo Ortega 02950, 4780000 Temuco, Chile; 5 Núcleo de Investigación en Bioproductos y Materiales Avanzados (BIOMA), Facultad de Ingeniería, Universidad Católica de Temuco, Ave. Rudecindo Ortega 02950, 4780000 Temuco, Chile

**Keywords:** Environmental sciences, Environmental chemistry, Environmental impact

## Abstract

One of the problems that most affect humanity today is the wastewater discharge into different water bodies. It was estimated that more than 7 million tons of wastewater are generated worldwide and are discharged into rivers, lakes, and reservoirs. Among the most dangerous wastewaters are those from inorganic chemistry research laboratories, mainly due to heavy metals. These problems have become a highly relevant topic, and numerous researchers have tried to design wastewater treatment systems that will deal more efficiently with heavy metals elimination. In this work, the synthesis, characterization, and evaluation of hydrated aluminium silicate were performed as alternative wastewater treatment from chemistry research and teaching laboratories. The compound obtained was $Al_2O_33SiO_2H_2O$, which was characterized by the determination of its physicochemical properties. These revealed a low density, very porous material, with low crystallinity, strong chemical resistance, a large surface area, and a high apparent ionic exchange capacity. Absorption kinetics studies of heavy metals in aqueous solutions, through more widespread models, have demonstrated that $Al_2O_33SiO_2H_2O$ has excellent properties as absorbents of this material. The amorphous hydrated aluminium silicate achieves a decrease in the concentration of all the metal ions studied, reducing them to discharge levels permissible.

## Introduction

The discharge of large wastewater volumes into the environment is currently one of the major environmental pollution concerns^1–4^. Among the more common and harmful pollutants are heavy metals, which produce a vast amount of emissions worldwide (just considering the industrial facilities of the European Union member countries, 3,598 tons of heavy metals were discharged into the water in 1 year)^5,6^. Discharges of wastewater from various industrial activities such as electroplating, paint production, plastics, metal materials, mining, some energy producers, and welding materials are the main sources of discharges of heavy metals content into the environment^7–10^. Thus the great importance of identifying, evaluating and knowing more about the effects of the species present in these residues in order to avoid present and future damage through responsible, safe, efficient, legal and low-cost wastewater management^11–14^.

The toxicity of metals depends on the chemical species in which it is part, the routes of administration, and the optimal concentration levels above which they are toxic^15,16^. Usually, results when an organism is subjected to an excessive concentration of the metal for an extended period, when it appears in a specific biochemical form or when the organism absorbs it by an unusual route^17,18^. Since both the deficiency and the elevated levels of many metallic species can lead to adverse effects for health and the environment; Various studies have been documented on the need for them to be discharged at adequate levels. From this problem on the protection of the environment and human health; Considerable attention has been paid to the development capable methods of reducing the concentration of metals in aqueous wastes^19,20^.

Many studies have been conducted to establish procedures or alternatives directed at reducing the presence of toxic metals in wastewater^21,22^. The residue treatment systems are categorized as physicochemical or primary, biological or secondary, and tertiary treatments^11^. The main techniques used in these systems are chemical precipitation, oxidation-reduction, ionic exchange, electrochemical treatments, membrane processes, solvent extraction, bio-adsorption, and adsorption onto adsorbent materials^3,6,23–28^.

Based on these wastewaters characteristics, one of the procedures studied is a primary treatment by metals precipitation adjusting the pH level, followed by a secondary adsorption treatment using clay as the adsorbent material^29^. This procedure has a limiting factor the secondary treatment is not sufficient, because it does not manage to reduce the heavy metals total amount present to allowable discharge levels^29^. This study does not report the behavior of the sorbent for Cr(III), Cd(II), and Hg(II) ions, which are commonly used in teaching laboratories and present high hazard and toxicity levels^29^.

Adsorbent materials have been widely used to achieve a decrease in the concentration of metals to permissible levels to be discharged^10,30^. Adsorption is very advantageous in terms of economy, working flexibility, sensitive operating conditions and costly disposal, efficiency, and right result in metal ion elimination^10,20^. In this sense, many materials of natural and synthetic origin have been studied. These materials include activated carbons, Clays, Zeolites, Fly ash, Titanium dioxide, bioadsorbents, metal oxides, as $Al_2O_3$, among others^31^. The physicochemical properties of these materials play an essential role in performance as adsorbents. Some characteristic properties including high cation exchange capacity, large surface area, chemical, and mechanical stability.

Aluminum silicate is a material that has excellent properties to be used as a sorbent and can be obtained from low-cost reagents such as silicate and Al. This material is crystalline compounds that, at a molecular level, are made up of very well defined planes and faces, characterized by their ability to disperse X rays. These materials are composed of regular crystals with a basic unit in the shape of a tetrahedron^28^. This tetrahedron consists of one central atom of silicon (*Si*) and four of oxygen (*O*) at the corners. Each silicon atom has four positive electric charges, and each oxygen atom has two negative charges. That is why each oxygen atom must use one of its charges to attract one of the silicon charges, thus saturating the central atom (*Si*). In contrast, the corners still have one negative charge to link to another atom with a positive charge. When this occurs, a chain of tetrahedrons can be formed with links such as (${-}O{-}Si{-}O{-}Si{-}O{-}$)^28^.

Aluminum compounds have been used as an absorbent for the removal of methylene blue, malachite green, and rhodamine-B from aqueous solutions from the textile wastewater^32^, the fly ash as aby-product generated during the coal combustion in thermal power plant^33^. Another silica compounds have been used to remove C.I. Basic Violet, Acid Orange 7, C.I. Reactive Black 5, C.I. Direct Blue 7 from the textile wastewater^32,33^. However, these compounds have also been used to remove heavy metals from laboratory-prepared solutions^34^ and wastewater^35^. Based on what was previously explained, our research group set the task of synthesizing and characterizing hydrated aluminium silicate as an alternative to removing heavy metals from wastewater.

## Results and discussion

This section presents the main results obtained from the synthesis of amorphous hydrated aluminium silicate as a sorbent of heavy metals and its possible use in the treatment of laboratory residues with a high content of these species. To this aim, a chemical–physical characterization of this silicate was made, establishing characteristics and properties that could allow us to evaluate both the sorption process *vis-à-vis* specific species and the conditions in which it could be used.

### Chemical characterization of aluminium silicate

The experiments performed in the laboratory revealed that amorphous hydrated aluminium silicate ($Al_22O_33SiO_2H_2O$) has a mass of 300.13 g/mol, of which 32.82% corresponds to $Al_2 O_3$, 58.16% to $SiO_2$, 5.10% to $H_2O$, 2.39% to *NaCl* and 1.52% to $Fe_2O_3$.

Another significant aspect of characterizing the material is its resistance to different aggressive media such as corrosive, acidic, basic, and oxidizing agents. The behavior of the material *vis-à-vis* these media allows us to evaluate the conditions in which it can be used without giving rise to chemical modification. This study shows that amorphous hydrated aluminium silicate is strongly resistant to acidic, basic, oxidizing and corrosive media, given that there is no appreciable mass variation in the material after being in contact with the different media over 24 h. This property allows it to be used unrestrictedly by the chemical features of the residue.

Other properties in the material characterization are to determine its heat of dissociation. We determined the ionic product at temperatures in order to calculate the enthalpy of the dissolution process. The results obtained from Eq. 11 allow us to propose a clear dependence between the ionic material product and the temperature (Eq. 1)

1 $$\begin{aligned} \begin{array}{cc} ln PI=C-\frac{\Delta H_d}{RT} 354.97T-35.244 &{} (R^2=0.967) \\ \\ -\frac{\Delta H_d}{R}=354.97 &{} \Delta H_d=-2.951\,\hbox {kJ/mol} \end{array} \end{aligned}$$

As was showed in the result derived from Eq. 1, the material dissociation process was exothermic. Therefore it will be favored at low temperatures. Given this result, its use at low temperatures is not recommended. Two of the fundamental experiments in the material characterization are the determination of the infrared spectrum and X-ray diffraction.

**Figure 1:**
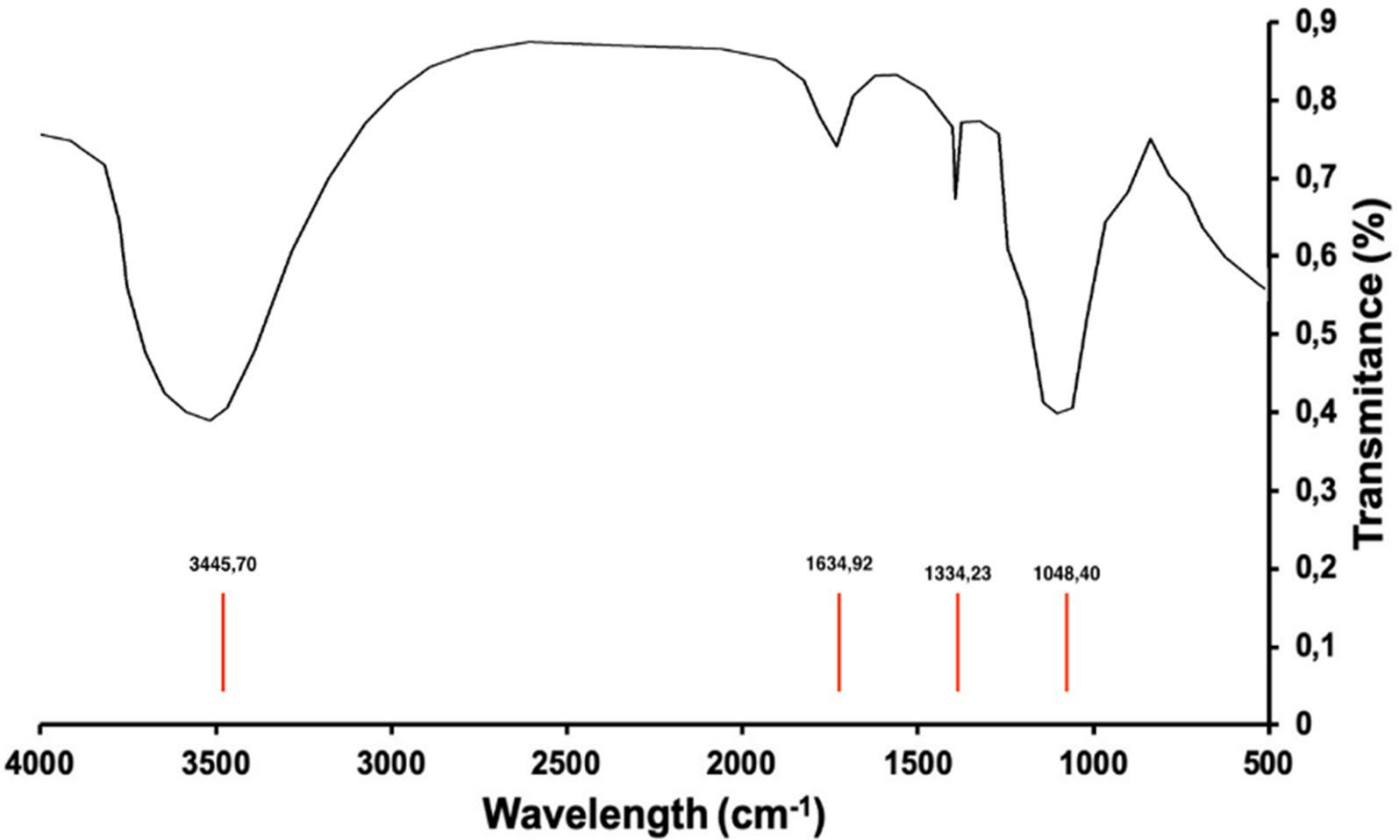
IR spectrum corresponding to amorphous hydrated aluminium silicate.

The infrared spectrum analysis (Fig. 1) confirms the presence of bands characteristic of metal silicates in the 514–1480 cm$^{-1}$ zone, referring to the different types of internal oscillations within the network ($O{-}Si{-}O$ y $Al{-}O{-}Si$)^36^. The 1634.92 and 3445.70 cm$^{-1}$ bands are attributed to structural water ($O{-}H$) ^37^.

**Figure 2:**
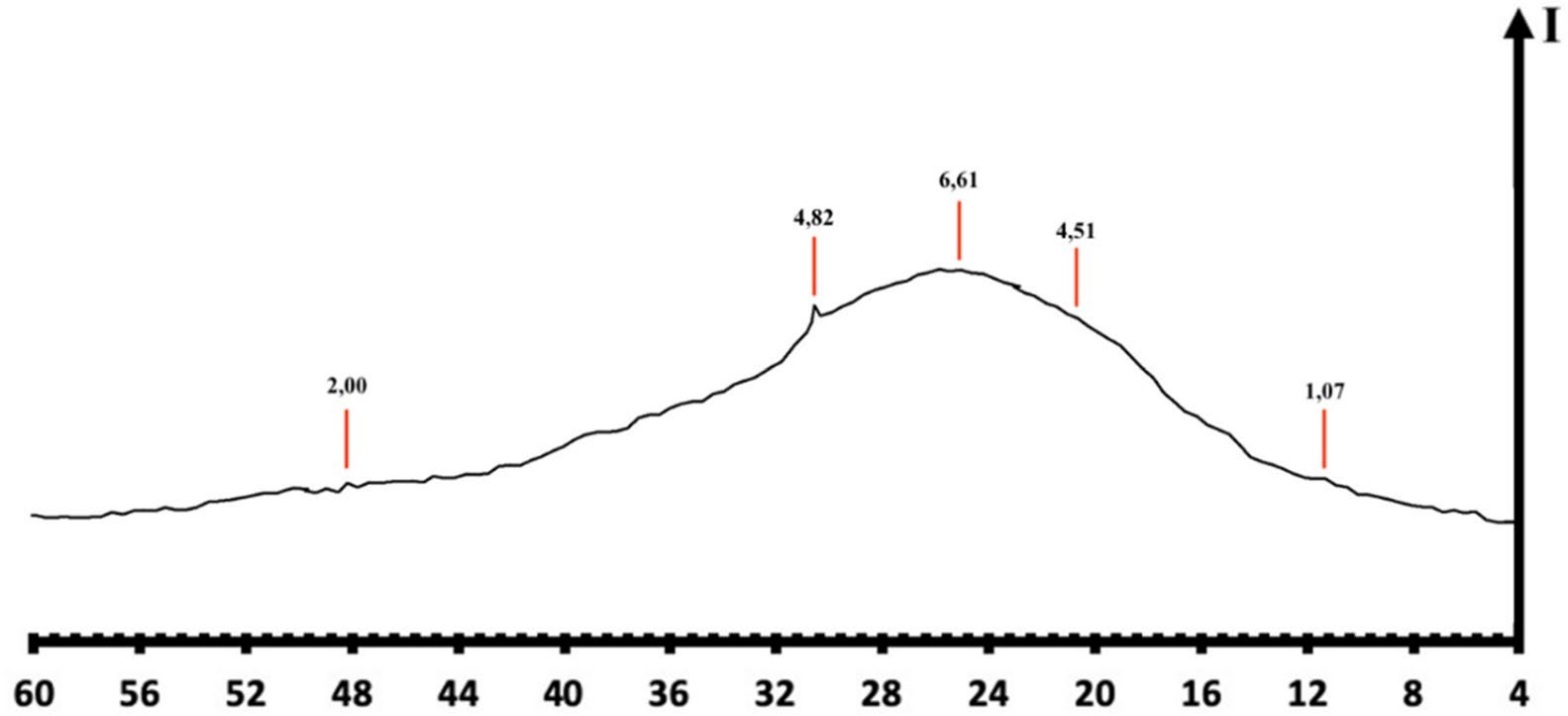
X-ray diffraction for amorphous hydrated aluminium silicate.

The X-ray diffraction record results provided evidence of low crystallinity of the product and its more excellent amorphous quality (Fig. 2), a property favoring adsorbent materials^38,39^.

### Physical characterization of aluminium silicate

The material physical characterization revealed a series of properties inherent to sorbents such as real density, apparent density, apparent density through entrapment, compressibility, porosity, flow rate, and tortuosity, which are shown in Table 1.

**Table 1:** Physical properties determined from hydrated aluminium silicate synthesized.

Properties	Value
Real density ($d_r$) (g/cm^3^)	$1.501 \pm 0.023$
Porosity (*P*) (%)	$86.7 \pm 0.5$
Apparent density ($d_a$) (g/cm^3^)	$0.203 \pm 0.029$
App. Dens by Entrapment ($d_{apa}$) (g/cm^3^)	$0.396 \pm 0.024$
Compresibility (*c*) (%)	$48.7 \pm 0.3$
Turtuosity ($d_t$) (g/cm^3^)	1.13
Flow rate ($V_f$) (m/s)	0

With these properties, it is possible to state that the material exhibits low density, high grain porosity, and that it can be compressed practically by 50%. Likely its flow rate equal to zero, we can say that it is a material with a large surface area, which could be charged and very porous, allowing it to adhere firmly to other surfaces without flow. These features are very favorable for adsorbent materials.

The specific surface area was another parameter established, which was determined by the methylene blue method. The results were in a dependence that adjusts to the Langmuir model when representing $\frac{1}{q_e}$ versus $\frac{1}{C_t}$^40^.

**Figure 3:**
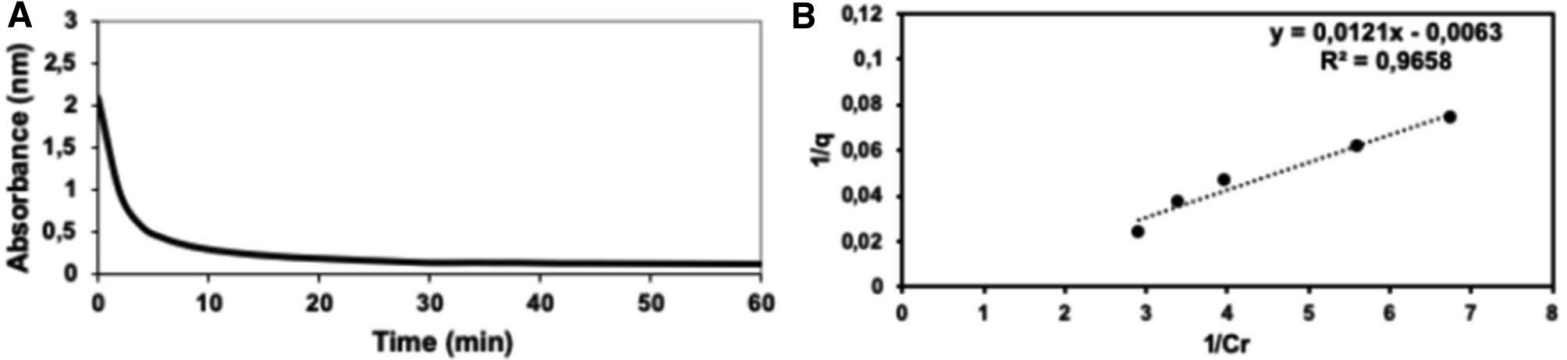
Graphical representation of the kinetic behavior of amorphous hydrated aluminium silicate: A- kinetic model; B-Langmuir model.

As shown in Fig. 3A,B, the equation of the line obtained was $$y = 0.012x-0.0063$$, $$R^2=0.9658$$. Once the process was adjusted to the Langmuir model the specific surface area of the material can be determined by the methylene blue molecule ($$C_{16}H_{18}N_3S)Cl$$ (sub-index 2 in Table 2) or the ionic species ($$C_{16}H_{18}N_3S)^+$$ thereof, making it possible to establish important parameters related to the specific surface area (Table 2).

**Table 2:** Properties determined by the methylene blue method.

Surface area (methylene blue)	$A_1=467.21$ Å	$A_2=507.20$ Å
Specific surface area (m^2^/g)	$S_1=177.02$	$S_2=192.17$
Mean pore radius (cm)	$r_1=6.52$	$r_2=6.20$
Mean pore volume (cm^3^)	$V_{p_1}=0.57$	$V_{p_2}=0.57$
Hollow fraction	$F_{h_1}=0.42$	$F_{h_2}=0.42$

As shown in Table 2, the material presents acceptable values in these parameters, which characterize the good properties of amorphous aluminium silicate as a sorbent^41,42^.

### Kinetic study of the heavy metal absorption

The analysis of the different kinetic models was performed by the least-squares statistical method using the RStudio programme^43,44^. The results can be evaluated through the correlation coefficient ($R^2$) of the different kinetic and thermodynamic models studied for each ion shown in Table 5.

**Table 3:** Correlation coefficient ($R^2$) of the kinetic models performed.

Ions	Pseudo first order	Pseudo second order	Elovich model	Intraparticle diffusion
$Pb^{2+}$	**0.91**	0.37	0.83	**0.91**
$Cd^{2+}$	0.61	**0.99**	**0.97**	0.89
$Co^{2+}$	0.73	**0.98**	0.85	**0.89**
$Cu^{2+}$	**0.99**	**0.91**	**0.96**	**0.97**
$Hg^{2+}$	0.88	**0.96**	0.86	**0.93**
$Ni^{2+}$	**0.98**	**0.98**	0.84	0.89
$Zn^{2+}$	0.74	**0.87**	0.76	**0.95**
$Cr^{3+}$	**0.88**	**0.99**	0.41	0.84
$Cr_2O_7^{2-}$	**0.99**	0.26	**0.99**	**0.99**

The numbers in bold correspond with the best adjustment to the studied models.

As shown in Table 3, the sorption process of $Pb^{2+}$ adjusts, preferably to pseudo-first-order kinetics and the intraparticle diffusion model. This result leads us to think that the process of lead sorption should be physical, with the formation of a monolayer on a heterogeneous surface. Intraparticle diffusion is an aspect to be taken into account. Adsorption presents a pseudo-first-order rate constant of 0.058 min$^{-1}$ with a mean lifetime of 11.01 min and a total lifetime of 12 min. The product presents a sorption capacity of 1.02 g per gram of adsorbent.

In the case of cadmium(II), the sorption process adjusts to pseudo-second-order kinetics, unlike $Pb^{2+}$. The result is characteristic of physical absorption processes with the monolayer formation on a heterogeneous surface. The process is governed by the intraparticle diffusion of the adsorbate by adsorption–desorption in different sites. Adsorption presents a pseudo-second-order rate constant equal to 0.1944 mg/g min, with a mean lifetime of 0.217 min and an initial adsorption rate of 105.22 mg/g min. The product has a sorption capacity of 0.623 g per gram of sorbent in a 13 min time.

In terms of cobalt(II) the process adjusts to pseudo second order kinetics, the same as $Cd^{2+}$. The cobalt absorption process should be physical. It could be governed by the intraparticle diffusion of the adsorbate by adsorption–desorption in different sites, with the formation of an adsorbate layer on a heterogeneous surface. Adsorption presents a pseudo-second-order rate constant equal to 0.01944 mg/g min, with a mean lifetime of 4.14 min and an initial adsorption rate of 0.242 mg/g min. The product presents a sorption capacity of 0.99 g per gram of sorbent in a 30 min time.

The case of copper(II) is quite distinct. The element has a sorption process that was adjusted to pseudo-first-order and intraparticle diffusion kinetic models. Thus establishing that its adsorption should be physical, where an aspect to be taken into account is the diffusion of the adsorbate, perhaps more localized on one single site. The metal adsorption by the amorphous aluminium silicate presents a pseudo-first-order rate constant equal to 0.0536 min$^{-1}$ , with a mean lifetime of 12.93 min. Copper has a sorption capacity of 0.064 g per gram of sorbent in a 60 min time.

In the mercury case, adsorption was adjusted to pseudo-second-order and intraparticle diffusion kinetic models. It was established that its sorption should be physical intraparticle diffusion of the adsorbate. It should be taken into account, through adsorption–desorption in different sites. This fact occurs on a heterogeneous surface with the monolayer formation. Mercury adsorption presents a pseudo-second-order rate constant equal to 0.0795 mg/g s, with a mean lifetime of 12.03 min and an initial adsorption rate of 0.867 mg/g s. The product presents a sorption capacity of 0.61 g per gram of sorbent in a 30 min time.

In terms of nickel, sorption adjusts kinetically to pseudo-first and pseudo-second-order models. The adsorption of this metal by the synthesized material should be physical. The intraparticle diffusion of the adsorbate has to be considered with the possible multilayers formation on a heterogeneous surface. Adsorption presents a pseudo-second-order rate constant equal to 9.10–5 g/g min, with a mean lifetime of 3.33 min and an initial adsorption rate of 0.300 g/g min. The product presents a sorption capacity of 0.69 g per gram of sorbent in a 30 min time.

Concerning zinc, its absorption process adjusts to the pseudo-second-order and intraparticle diffusion kinetic models, where the adsorption should be physical, and the intraparticle diffusion of the adsorbate via adsorption–desorption in different sites is an aspect to be taken into account together with the monolayer formation on a heterogeneous surface. The adsorption presents a pseudo-second-order rate constant equal to $1.9*10^{-4}\,\hbox {g/g}\,\hbox {min}$, with a mean lifetime of 16.7 min and an initial adsorption rate of 0.06 g/g min. The product presents a sorption rate of 0.71 g per gram of sorbent in a 30 min time.

The chromium(III) sorption process adjusts to pseudo-second-order kinetics so that it can be said that the process should be physical with the monolayer formation on a heterogeneous surface, governed by the diffusion of the adsorbate via adsorption–desorption in different sites. Adsorption presents a pseudo-second-order rate constant equal to 2.54 g/g min, with a mean lifetime of 0.31 min and an initial adsorption rate of 3.54 g/g min. For this element, the material presents a sorption capacity of 1.13 g per gram of sorbent in a 27 min time.

Chromium(VI) presents more complex kinetics than the other elements, perhaps associated with the tremendous structural difference of the species of which it is a part ($Cr_2O_7^{2-}$), that presents a negative charge density ($\delta ^-$). Its adsorption is not very defined from a physical or chemical point of view, because during the first 24 min its sorption adjusts to the Elovich model (chemical), with a possible species transformation. The adsorption also presents a certain degree of adjustment to the pseudo-first-order (physical). This effect would be associated with an interaction presumably with the Si(IV) and/or the Al(III), that are part of the aluminosilicate network and/or with the K(I) present in the $K_2Cr_2O_7$ salt used. That could be adsorbed on the surface of the material and interact physically with these species with charge density ($\delta ^+$). Therefore both types of interactions with the material could exist simultaneously in similar proportions. After the 24 min has elapsed, the adsorbate experiences 50% desorption, which could be associated with the part adsorbed more weakly (the physical adsorption) as a result of Van der Waals forces (weaker molecular interactions). In this case, it must be weaker than the others because of a result of the material surface characteristics.

The analysis of different kinetic model’s adjustments, Pb(II) and Cu(II) shown pseudo-first-order kinetics and Cd(II), Co(II), Hg(II), Ni(II), Cr(III) and Zn(II) adjust to pseudo-second-order kinetics. The latter group presents preferably physical type sorption, with low lifetime spans and adsorption governed by the diffusion of the adsorbate via adsorption/desorption in different sites. In all cases, diffusion is a crucial aspect to be considered. An exception is Cr(III), which has a much higher charge/radius relation than the others, which would seem to be more determinant in its adsorption than diffusion. In the case of $Cr_2O_7^{2-}$ the kinetics are more complex, with desorption occurring after 24 min, with adjustments initially favoring an Elovich and secondly a pseudo-first-order model, therefore perhaps experiencing a simultaneous chemical and physical sorption^45^.

**Figure 4:**
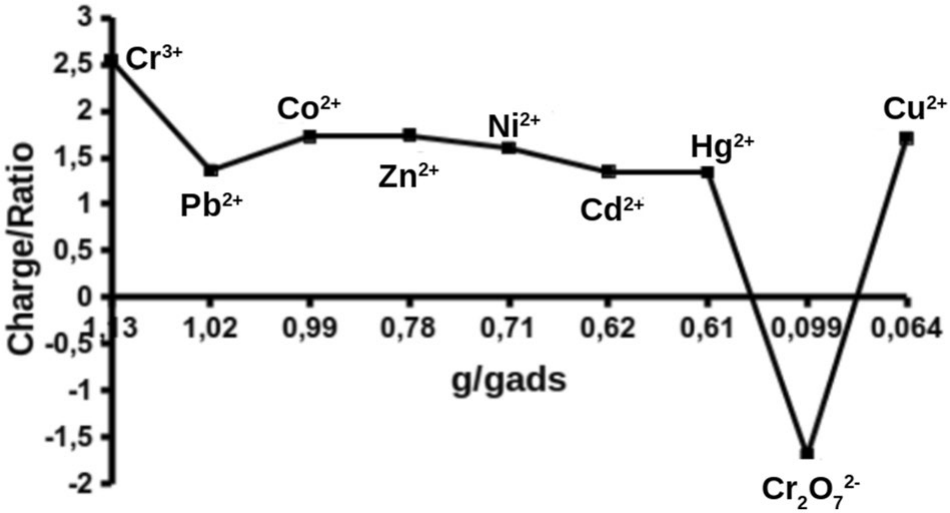
Charge/radius relation versus sorption capacity.

In terms of the differences existing in the sorption capacity, we observe that: $Cr(III)> Pb(II)> Co(II)> Zn(II)> N i(II) \ge Cd(II) \approx Hg(II)> Cr(V I) > Cu(II)$. This order is very similar to the one established with effective diffusion (Def) and the charge/radius relation of the metal (Fig. 4) so that there would seem to be a marked influence of these properties on the sorption capacity of each adsorbate, except for Cr(III) whose charge/radius relation could be more determinant, and for $HgCl_2$, whose covalent character limits its dissociation and diffusion.

Figure 4 shows that as the charge/radius relation declines, there is a decrease in sorption capacity. This is consistent with the sorption type that is established, which is preferably physical^45^. Only Pb(II), Cu(II), $Cr_2O_7^{2-}$ and to a lesser degree, Zn(II) present a significant deviation. The first is consistent with a phenomenon existing in aqueous systems related to the greater polarisability and lower enthalpy of this species concerning the others^36^, which allows them to diffuse more easily (Fig. 4) and get closer to the surface of the solid and interact better with it. For this reason, lead appears to have the second maximum adsorption despite having the lowest charge/radius relation, which is to be expected about different studies^46,47^.

Zn(II) is consistent with the same phenomenon observed in Pb(II) because it has lower hydration enthalpy than the others (though not as pronounced as Pb(II)). Its coordination components in an aqueous solution have a tetrahedral structure. Perhaps allowing it to display lower steric hindrance is revealed by its presenting a higher diffusion coefficient than Ni(II), which should have a higher adsorption capacity than Zn(II).

In the Cu(II) case, another phenomenon in its coordination compounds is present, the Jahn–Teller effect^48^. This effect is associated with an elongation of the links directed along the length of the z-axis of the coordinate system, which provokes a deformation of the octahedron that could hinder its movement within the aluminosilicate network (this species has the highest adsorption rate and lowest effective diffusion coefficient except for Hg). It could, therefore, have less interaction with the entire active surface of the material and a lower degree of adsorption. The case of $Cr_2O_7^{2-}$ was already explained above^49^.

### Study of alternative treatment for heavy metal removal

This study encompassed a primary treatment based on the precipitation of the metals in the form of a sparingly soluble substance with $Ca(OH)_2$, followed by a secondary treatment based on the sorption of the species with the amorphous hydrated aluminium silicate. The Table 4 shows the results obtained in the different treatment stages; where *C* o is the concentration of the species before treatment, $C_2$ after precipitation with calcium hydroxide and $C_3$ after treatment with the amorphous hydrated aluminium silicate.

**Table 4:** Results of the material behavior in the presence of a mix of these ions.

Species	$C_0$ (mg/L)	$C_2$ (mg/L)	$C_3$ (mg/L)	Removal (%)	$q_e$ (mg/g)	t (min)
$Cr^{3+}$	311.75	5.3	0.05	99.01	5.01	10
$Pb^{2+}$	432.5	2.72	0.22	91.84	2.38	10
$Co^{2+}$	335.25	15.2	0.107	99.30	14.3	10
$Zn^{2+}$	698.25	27.7	0.41	98.51	25.90	20
$Ni^{2+}$	418.00	3.16	0.224	92.91	2.80	10
$Cd^{2+}$	504.5	0.42	0.04	90.48	0.36	10
$Hg^{2+}$	331.27	0.08	0.009	89.55	0.08	20
$Cu^{2+}$	383.25	0.661	0.054	91.83	0.58	20
					Total	51.46

These results show that aluminium silicate presents high removal percentages and is useful in the treatment of a mix of several ions, achieving a reduction in the concentration of all species and reducing them to allowable discharge levels after a mere 20 min contact using only 1.05 g of the material.

The design of more efficient wastewater treatment systems is a crucial matter for life on our planet. The discharge of growing concentrations of heavy metals to the environment has caused a mass exposure of different animals to these elements, including humankind. In this work, we have synthesized hydrated amorphous aluminium silicate, which proves to have the ability to remove heavy metals, reduce them to permissible discharge levels, and be used to treat residues with these species present.

This material displays good chemical and physical properties as a metal ions sorbent. It can be used unrestrictedly following the residue chemical characteristics given its strong resistance to the different media. The kinetic studies show adsorption that should preferably be physical, where intraparticle diffusion is an aspect to be taken into account, together with the formation of one layer on a heterogeneous surface. Exceptions to the above are $Cr_2O_7^{2-}$, which could present both physical and chemical adsorption and Ni(II), which adjusts to the BET model, applicable to the formation of multilayers. As an adsorbent of the ions understudy, amorphous hydrated aluminium silicate presents a good sorption capacity in working conditions, with $Cr(III)> P b(II)> Co(II)> Zn(II)> N i(II)> Cd(II) \approx Hg(II)> Cr(V I) > Cu(II)$, for which diffusion and the charge/radius relation of the species should have a major influence. Amorphous hydrated aluminium silicate manages to reduce the concentration of all the metal ions understudy to allowable discharge levels.

**Table 5:** Maximum adsorption capacity of metal ions by different material the natural and synthetic origin.

Adsorbent material	$q_e$ (mg/g)	t (min)	References
Alternanthera philoxero	257.1 ($Pb^{2+}$)	180	Yang et al.^50^
Miscanthus	13.2 ($Cd^{2+}$)	60	Kim et al.^51^
Sugar cane bagasse	135.5 ($Pb^{2+}$)	90	Inyang et al.^52^
Dairy-Manure	140.9 ($Pb^{2+}$)	240	Cao et al.^53^
Hickory wooda	28.12 ($Cd^{2+}$)	60	Wang et al.^54^
Pig manure	230.7 ($Cd^{2+}$)	10	Kołodyńska et al.^55^
Cow manure	118.4 ($Cd^{2+}$)	10	Kołodyńska et al.^55^
Biochar (CS0)	289.3 ($Pb^{2+}$)	15	Chen et al.^56^
Biochar (CS0)	171.9 ($Cd^{2+}$)	15	Chen et al.^56^
graphene sand composite	28.59 ($Cr^{2+}$)	90	Dubey et al.^57^
Lignocellulose/Montmorillonite Nanocomposite	94.8 ($Ni^{2+}$)	40	Wang et al.^58^
Palygorskite	8.8 ($Co^{2+}$)	180	Yuanming et al.^59^
xanthate-modified magnetic chitosan	76.9 ($Pb^{2+}$)	120	Jianlong et al.^60^
xanthate-modified magnetic chitosan	34.5 ($Cu^{2+}$)	80	Jianlong et al.^60^
xanthate-modified magnetic chitosan	20.8 ($Zn^{2+}$)	110	Jianlong et al.^60^
Paenibacillus polymyxa bacteria	1.602 ($Cu^{2+}$)	120	Ravikumar et al.^61^
$Al_2O_3$	41.2 ($Pb^{2+}$)	60–90	Mahdavi et al.^62^

As it is shown in Table 5, and according to the results obtained regarding the removal of heavy metals (Table 4) the amorphous aluminum silicate synthesized by our group pursues an excellent ability to remove metals weigh yourself compared to other materials. Removal times were lower than those found in the literature, which leads us to think that our material may be a good alternative for removing heavy metals in a liquid sample.

## Methods

### Synthesis of aluminium silicate

The aluminium silicate was synthesized using the sol–gel method described in the literature^63^. This is the most commonly feasible and straightforward procedure used to obtain at a laboratory level^64,65^. For the material synthesis under study in this work, we used the following chemical reaction:

2 $$\begin{aligned} 2AlCl_3{(ac)} + 3 Na_2O*3SiO_2*H_2O(ac)\longrightarrow Al_2O3*3SiO_2*H_2O(s) + 6NaCl(ac) \end{aligned}$$

For the synthesis of 100 g of aluminium silicate, we used 1 L of $AlCl_3$ at a concentration of 0.66 M and one liter of sodium silicate at a concentration of 0.99 mol/L. This solution was left to stand for 24 h at a temperature of 30 °C, then filtered and washed with deionized water until verifying that the mother liquor had reached a pH 7 level. It was subsequently dried at 80 °C, crushed and sieved in order to obtain a particle between 0.125 and 0.200 mm; following with its characterization.

### Characterization of aluminium silicate

The material synthesized was characterized in the laboratory by a series of procedures that allowed us to determine the material chemical structure, impurities and some physicochemical properties of the obtained product, which are described below.

#### Chemical characterization of aluminium silicate

The chemical characterization of aluminium silicate was performed to determine the material chemical behavior. The aluminium silicate was placed in contact with different media (an acid medium made up of a solution of HCl(ac) at 33%, a corrosive medium composed of a solution of NaCl(ac) (0.0085 M) and exposed to an oxidizing agent, in our case a solution of NaOCl(ac) at 10%). In each case, after 24 h of treatment, a measurement was made of the mass loss of the synthesized material.

Another measured parameter for the amorphous aluminium silicate characterization was the determination of the heat of dissociation and the ionic product. The heat of dissociation was determined to subject the synthesized compound to different temperatures, which allowed us to establish the dissociation enthalpy ($\Delta H_d$) and evaluate the dissociation process in water. To this the following equation was used^66,67^.

3 $$\begin{aligned} LnPI=C-\frac{\Delta H_d}{RT} \end{aligned}$$

where *PI* corresponds to the material’s ionic product, which was obtained as described below. On the basis of Eq. 3, we made a graphic representation of *PIvs*1/*RT*, resulting in a slope (*m*) ($m =-\Delta H_d /RT$). This slope value allows us to obtain $\Delta H_d$ using the following equation:

4 $$\begin{aligned} \Delta H_d=(mRT) \end{aligned}$$

where $\Delta H_d$ correspond to the enthalpy variation, *R*, the gas constant and *T* , the temperature in Kelvin degree. The ionic product of the material was determined by the following chemical reaction:

5 $$\begin{aligned} Al_2(SiO_3)_3(s) \leftrightarrows 2Al^{3+}(ac)+3SiO_3^{2-} \end{aligned}$$

From this chemical reaction we can calculate the ionic product of the material via the following expression:

6 $$\begin{aligned} PI=\left[ Al^{3+}\right] ^2 *v\left[ SiO^{2-}\right] ^3 \end{aligned}$$

The infrared analysis is another characterization methods that are commonly used in synthesized compounds^68,69^. In this study, we followed the standard procedure reported in the literature^68,69^, using a Phillips FTIR model PV-9512 spectrophotometer and KBr tablets.

#### Physical characterization of aluminium silicate

The physical characterization of the material synthesized in the laboratory included determining physical parameters, such as real and apparent density. The apparent density was realized using entrapment, hardness, grain porosity, compressibility, tortuosity, and flow rate. For all these determinations, ten replications of each sample were performed.

The real or pycnometric density (dreal) was determined by means of the pycnometric method, using a 50 ml Weld-type pycnometer at 25 °C. The technique consists of using a 0.001 g precision scale to weigh the empty pycnometer, then filled with petroleum ether and 1g of the product. The ($d_{real}$) was calculated using the following formula^66^:

7 $$\begin{aligned} d_{real}=\frac{m_p}{\frac{V_{Pic}-m_{sol}}{d_{sol}}} \end{aligned}$$

where $m_p$ is the dust mass (g),$V_{Pic}$, the volume of the pycnometer (ml),$m_{sol}$, the solvent mass (g) and $d_{sol}$ the solvent density (g/ml) at the experiment temperature ($25^{\circ }C$). The material apparent density was calculated by the volume displacement of the synthesized compound^66^:

8 $$\begin{aligned} d_a=\frac{\sum m}{\frac{n}{V}} \end{aligned}$$

where $d_a$ is the apparent density (g/ml), $V_{Pic}$, the dust mass (g), *n*, the number of replications and *V* the volume of the test tube (ml).

Grain porosity is a measure of the roughness and capillarity of a given surface^70^. The determination of this parameter can be quite complex, but a simple way is to link the porosity to the material density using Martin’s equation^71^:

9 $$\begin{aligned} P=\left[ 1-\frac{d_a}{d_{real}}\right] *100 \end{aligned}$$

where P is the porosity (%), $d_{real}$, the real density (g/ml) and $d_a$ is the apparent density (g/ml), which was calculated similar to the real density, with the difference that the material was sieved using a German-made MLW sieve shaker.

Another aluminium silicate properties that we measured in this work was compressibility. This parameter is defined by the property displayed by solids to reduce the volume they occupy through an external force such as vibration, pressure, or agitation^72^. We obtained this property using the following equation^73^:

10 $$\begin{aligned} c=\left[ 1-\frac{d_a}{d_{apa}}\right] *100 \end{aligned}$$

where *c* is the compressibility (%), $d_a$ , the apparent density (g/cm$^3$) and $d_{apa}$, the apparent density by means of entrapment (g/cm$^3$).

One of the significant properties measured to characterize the material was the specific surface area (*S*). To measure this parameter, methylene blue is used as a benchmark^74^. Before this, a kinetic study was carried out to determine the compound’s maximum sorption time. This was performed by placing the methylene blue (15 mg/L) in contact with the silicate, previously dried at 200 $^{\circ }$C. The absorbance values were thereby determined at 660 nm, at intervals of 2 min by visible, ultraviolet spectroscopy, using a model PV-9512 FTIR spectrophotometer.

After establishing the time in which maximum sorption was obtained, a thermodynamic study was conducted based on methylene blue samples at known concentrations (0.6, 1.2, 1.5, 1.8, 2.4, 3.0, 4.5, 7.5. 10.5 and 15.0 mg/L) placed in contact with 0.1 g of aluminium silicate for 1 h. The absorbance levels were then evaluated by the Langmuir model. If this model is valid, it can be considered that adsorption occurs homogeneously over the entire surface, making it possible to determine the monolayer mass, from which the specific surface area is obtained using the following equation^74^:

11 $$\begin{aligned} S=\frac{q_ebN_aA_m10^{-20}}{M_a} \end{aligned}$$

where *S* is the specific surface area (m$^2$/g), $q_e$, the number of milligrams absorbed per gram of solvent (mg/g), $A_m$, the surface area of the methylene blue ($\AA {}^2$) and the monolayer mass (g), the methylene blue surface area was established using standard procedures described in the literature^40^.

### Kinetic study of heavy metals absorption

For the kinetic study of the absorption of the heavy metals, the synthesized material, previously dried at $200^{\circ }C$, was placed in contact with a known metal concentration solution (Table 6). Each sample was subjected to the exposure of individual metal, and we measured the absorption capacity (Table 6).

**Table 6:** Concentration, product mass and maximum sorption time values used for the kinetic analysis.

Heavy metals ions	Concentration (g/L)	Absorbent mass (g)	Time (min)
$Pb^{2+}$	0.30	0.1	12
$Cd^{2+}$	0.10	0.5	13
$Co^{2+}$	0.10	0.1	30
$Cu^{2+}$	1.0	0.1	60
$Hg^{2+}$	0.30	0.1	11
$Ni^{2+}$	1.46	0.5	30
$Zn^{2+}$	0.02	0.1	30
$Cr^{3+}$	0.30	0.1	27
$Cr_2O_7^{2-}$	1.54	0.86	24

The concentration values were determined at different time intervals using the atomic absorption spectroscopy method. The samples for atomic absorption spectroscopy analysis were contracted to an accredited laboratory of the Center for agricultural research of the Universidad Central “Marta Abreu” in Las Villas, Villa Clara province, Cuba.

These values make it possible to establish maximum sorption time and evaluate the results by the Elovich, intraparticle diffusion, pseudo-first, and pseudo-second-order kinetic models. The equations describing the models mentioned above are outlined in the following table (Table 7).

**Table 7:** Kinetic models of heavy metal absorption used in this study.

Models	Equations	Variable descriptor
Pseudo first order	$ln(q_e-q_r)=ln(q_e-k_1t)$ $t_{1/2}=\frac{ln2}{k_1}$	Dependence is established between $ln(q_e-q_t)$ versus *t* where $q_e$ is the amount in grams of solute adsorbed per gram of adsorbent at equilibrium (mg/g); $q_t$ represents the grams of solute absorbed per gram of absorbent over time (mg/g); *t* is the time in min, $k_1$ is the pseudo first order absorption rate constant ($min^{-1}$) and $t_{1/2}$ is the mean lifetime in min
Pseudo second order	$\frac{1}{q_t}=\frac{1}{k_2q_e^2}$ $t_{1/2}=\frac{1}{q_ek_2}$ $h_2=k_2q_e^2$	Dependence is established between $1/q_t$ versus *t*, where $q_e$ is the amount in grams of solute adsorbed per gram of adsorbent at equilibrium (mg/g); $q_t$ represents the grams of solute absorbed per gram of absorbent over time (mg/g); $h_2$ is the initial absorption rate (mg/gmin); $t_{1/2}$ represents the mean lifetime in min, and $k_2$ is the initial rate constant
Intraparticle diffusion	$q_t=kt^{1/2}+C$	Dependence is established between $q_t$ versus *t*, where $q_t$ is the amount of solute adsorbed per gram of absorbent over time (mg/g); $t^{1/2}$ represents the time variable in min; *k* is the intraparticle diffusion rate constant and *C* is a constant
Elovich model	$q_t=\alpha + \beta lnt$	Dependence is established between $q_t$ versus *lnt*, where $q_t$ represents the milligrams of solute adsorbed per gram of adsorbent over time (mg/g); $\alpha$ is the initial sorption rate (mg/min); $\beta$ is the sorption constant (mg/min) and *t* is the time in min

### Statistical procedure

The data obtained in the laboratory (10 replicates for each sample analyzed) were processed statistically employing the Kolmogorov–Smirnov^75–77^ and Bartlett^78^ tests to verify normality and homogeneity of variance. The correlation analyses were conducted using the Pearson simple linear correlation matrix^79^ and the Spearman rank correlation test^79^, both for a reliability level of 95%. All these statistical tests are included in the R statistical package version 3.6.2^43,44^.
